# ‘Painting my pain’: the use of pain drawings to assess multisite pain in women with primary dysmenorrhea

**DOI:** 10.1186/s12905-022-01945-1

**Published:** 2022-09-07

**Authors:** Jéssica Cordeiro Rodrigues, Mariana Arias Avila, Felipe Jose Jandre dos Reis, Roberta Moraes Carlessi, Amanda Garcia Godoy, Guilherme Tavares Arruda, Patricia Driusso

**Affiliations:** 1 Women’s Health Research Laboratory (LAMU), Physical Therapy Department, UFSCar, São Carlos, SP Brazil; 2 Study Group on Chronic Pain (NEDoC), Laboratory of Research on Electrophysical Agents (LAREF), Department of Physical Therapy, Universidade Federal de São Carlos (UFSCar), São Carlos, SP Brazil; 3 Physical Therapy Department, Federal Institute of Rio de Janeiro (IFRJ), Rio de Janeiro, Brazil

**Keywords:** Data accuracy, Dysmenorrhea, Psychometrics, Patient reported outcome measures

## Abstract

**Background:**

To verify the use of pain drawing to assess multisite pain in with primary dysmenorrhea (PD) and to assess its divergent validity, test–retest reliability, intra- and inter-rater reliability and measurement errors.

**Methods:**

Cross-sectional study. Adult women with self-reported PD three months prior to the study. Women answered the Numerical Rating Scale (NRS) and the pain drawing during two consecutive menstruations. The pain drawings were digitalized and assessed for the calculation of total pain area (%). Intra- and inter-rater reliability and the test–retest reliability between the first and the second menstruations were assessed with the intraclass correlation coefficient (ICC). Measurement errors were calculated with the standard error of measurement (SEM), smallest detectable change (SDC) and the Bland–Altman plot. Spearman correlation (rho) was used to check the correlation between the total pain area and pain intensity of the two menstruations.

**Results:**

Fifty-six women (24.1 ± 3.1 years old) participated of the study. Their average pain was 6.2 points and they presented pain in the abdomen (100%), low back (78.6%), head (55.4%) and lower limbs (50%). All reliability measures were considered excellent (ICC > 0.75) for the total pain area; test–retest SEM and SDC were 5.7% and 15.7%, respectively. Inter-rater SEM and SDC were 8% and 22.1%, respectively. Correlation between total pain area and pain intensity was moderate in the first (rho = 0.30; *p* = 0.021) and in the second menstruations (rho = 0.40; *p* = 0.002).

**Conclusion:**

Women with PD presented multisite pain, which could be assessed with the pain drawing, considered a reliable measurement.

## Introduction

Primary dysmenorrhea (PD) is a common condition among women of reproductive age and is defined as menstrual pain of uterine origin, in the absence of any associated pelvic or gynecological affection [1]. It has been postulated that PD-related pain is caused by the increased release of prostaglandins during endometrial desquamation, accompanied by ischemia and hypoxia of the myometrium [2]. PD-related pain can lead to limitation in physical and social activities [3], absenteeism from work and school and decreased quality of life [1, 2].

PD-related pain often occurs in the pelvic region or lower abdomen, and can be associated with fatigue, nausea, insomnia, headache, mood changes and gastrointestinal symptoms [1]. Although pain can be felt in other body regions (e.g., lower back, legs and head), generally, pain intensity of pelvic/abdominal pain is the only one assessed, as it is considered the most frequent symptom [3]. Nonetheless, women with PD may have pain intensity variation in different parts of the body and, in this sense, the pain drawings can help the health care professionals in the assessment of the subjective location [4] and pain distribution [5].

Pain drawings often provides information about patient’s diagnosis, can improve guidance regarding the most appropriate intervention and can be used to track recovery and illness trajectory. Body diagrams can also offer an insight into the psychological impact of pain: distress and frustration are often marked with shading that is very dense, with longer lines that sometimes extend beyond the body [6]. Inadequately managed pain could lead to adverse physical and psychological patient outcomes for individual patients and their families [7]. In the last years, several validated, computerized and automatic methods to measure pain distribution has been described and tested for other types of pain [8–10].

However, the assessment using a precise and validated method to measure PD-related pain is still lacking. This study aimed to verify the use of pain drawing to assess multisite pain in with PD and to assess its divergent validity, test–retest reliability, intra- and inter-evaluator reliability and measurement errors. The study hypothesis was that we would be able to identify several pain areas as to characterize the pain as multisite pain and to check if the pain drawing can be considered as an adequate tool to assess PD-related pain, with good reliability.

## Methods

### Ethical issues and study disclosure

This is a cross-sectional clinimetric study, carried out in the Women's Health Research Laboratory, at the Federal University of São Carlos, and followed the STROBE checklist [11]. The study was approved by the institution's research ethics committee (CAAE 29372620.0.0000.5504, protocol: 3.955.984), and all methods were carried out in accordance that Declaration of Helsinki. All women included in the study agreed to participate by reading the Informed Consent Form and after clicking “I agree to participate in this study”. Due to the COVID-19 pandemic, this study was carried out entirely online, through messaging applications (WhatsApp®).

The study was disclosed on social media and networks and on the institution's website. From this disclosure, women who were interested in participating in the study contacted the researchers, who recorded the contacts and scheduled the initial assessment.

### Participants and sample size calculation

Nulliparous women, aged between 18 and 45 years and with self-report of menstrual pain in the last three periods were recruited. Women with a self-report of a previous diagnosis of pelvic diseases, such as endometriosis and uterine fibroids, were excluded. The sample size calculation considered an Interclass Correlation Coefficient (ICC) as the minimum acceptable reliability of 0.50. A minimum sample size of 28 body diagrams would be required to be assessed by each examiner to achieve the statistical significance for an alpha value set at 0.05 and with a minimum power of 90%.

### Procedures

This study was carried out between September 2020 and December 2020. The first assessment consisted of filling out a questionnaire containing sociodemographic and clinical data, with information about the menstrual cycle, use of medications and non-pharmacological methods to relieve dysmenorrhea. In this assessment, the probable date of the beginning of the participant's next menstruation was collected. Close to this date, the responsible researcher sent the pain drawing and the Numerical Rating Scale (NRS) electronically to each research participant. Participants were instructed to print the pain drawing for completion and then scan the answer to return it to the researcher.

The completion of the pain drawing and the NRS occurred according to the regions of menstrual pain of each participant during the menstruation of two consecutive menstrual cycles.

### Measurements

#### Numerical Rating Scale

The NRS was used to assess the intensity pain during the first three days of their menstruation. For this, the participants answered the question "How would you rate your menstrual pain" with response options between zero (absence of pain) and 10 (the worst pain imaginable). The NRS has adequate test–retest reliability (ICC ≥ 0.67) for the assessment of general pain [12].

### Pain drawing

To complete the pain drawing, study participants were instructed by scripted email. The utterance instruction to fill in the pain drawing was “In the images below, paint the areas affected by pain during the current menstrual period. The region as a whole must be painted. Do not circulate the area. Do not mark the body regions with an “X”. The instructions for completing the area completely were intended to allow it to be read correctly by the software. The period for completing the pain drawing was the first three days of menstruation.

**Fig. 1:**
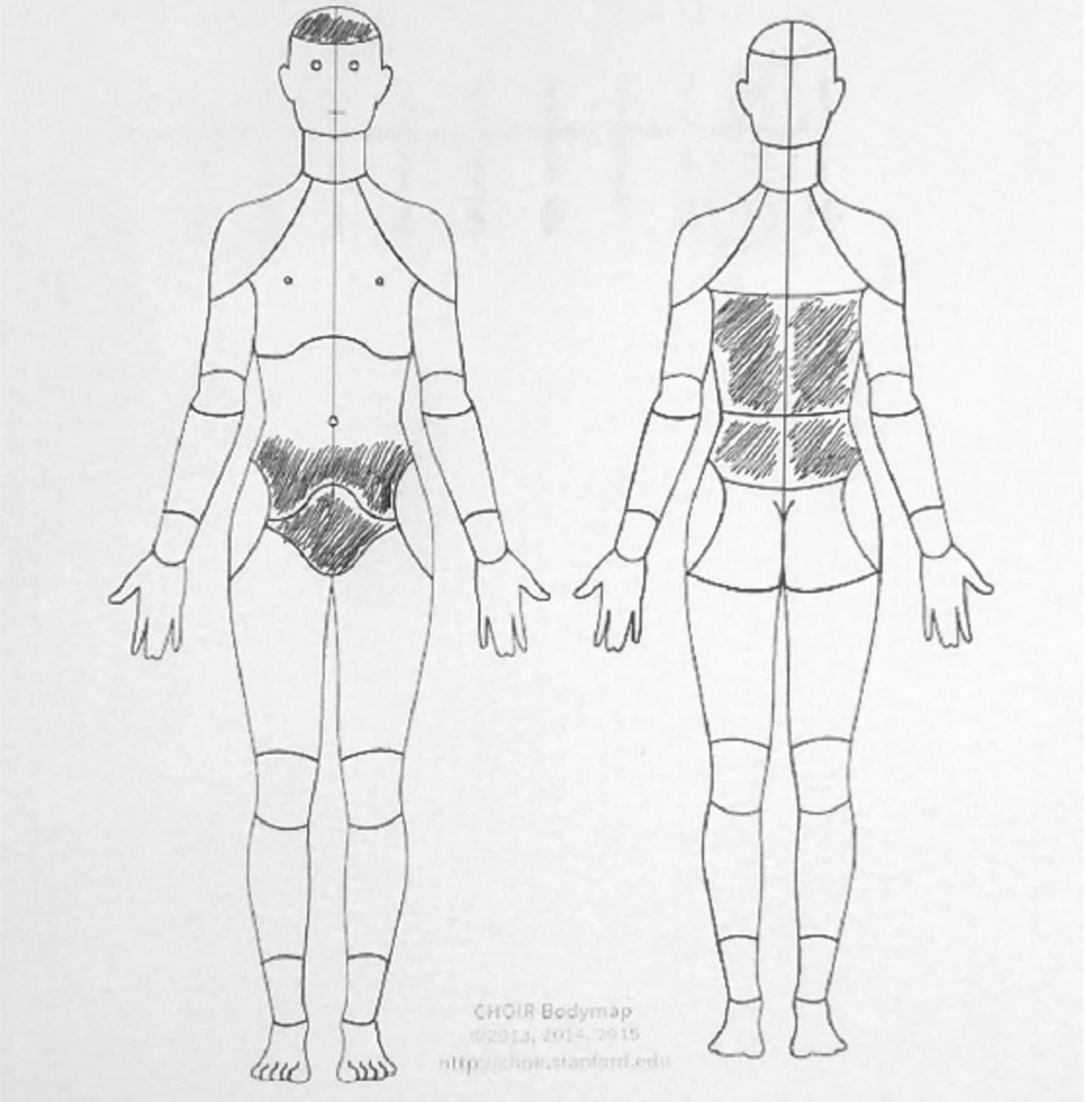
Example of pain drawing filled in by the participant

After completing the pain drawings by the participants, the images were photographed, digitized and stored in a JPEG image (Fig. 1) and then included in the ImageJ software.

### ImageJ software

The area related to pain during current menstruation painted by the study participants was calculated by the ImageJ software. The software can display, edit, analyze, process, save, and print images. It can also calculate the area and pixel value statistics, measure distances and angles, support standard image processing functions; and carry out spatial calibration to provide real world dimensional measurements in centimeters or millimeters [8].

### Procedures

Two examiners (E1 and E2) independently analyzed the areas painted on the images of each participant in the software (number of colored pixels within the body image). E1 is an experienced physiotherapist (specialized in Women’s Health Physical Therapy) and E2 physiotherapy student. E1 conducted the measurement of Body Map painted of each participant once (relative to the first menstruation), and E2 conduced analyzed the same Body Maps twice (relative to the first and second menstruations). A preliminary training practice session using the method was carried out to ensure familiarity with Body Map and Image J Software. To standardize the evaluation of images of different qualities, an evaluation of the total area and area of each region marked by the participant in pixels was performed, and then the percentage that each region represented of the total area was calculated.

### Data analysis

The intra- and inter-rater reliability analyzes of the first Body Map painted were performed using the intraclass correlation coefficient (ICC) with a two-way mixed-effect model and interaction for the absolute agreement between mean measurements, according to ANOVA assumptions. Excellent reliability was considered for ICC values above 0.75 [13]. We also calculate the concordance correlation coefficient (CCC) for inter-rater and test–retest evaluations, where values of 1 are considered perfect concordance [14]. The measurement errors of the inter-rater and test–retest evaluations were calculated by the Standard Error of Measurement (SEM), Smallest Detectable Change (SDC) at the individual level and analysis of the Bland and Altman graph. To calculate the SEM, the formula [SD_difference_/√2] was used, where SD difference is the standard deviation of the difference between the total body area of pain in the first and second menstruations [15]. The SDC was calculated by the formula [SEM*1.96*√2][13]. The limits of agreement (LoA) were calculated with the Bland and Altman plot using the formula [d- ± (1.96*SD_difference_)], where d- is the mean of the differences between the total body area of pain in the first and second menstruations [16].

To verify the correlation between total body area of pain and pain intensity measured by the NRS of the first and second menstruations – divergent validity, Spearman's correlation coefficient (rho) was used. Value of rho > 0.5 indicates strong correlation; 0.3–0.5, moderate correlation and rho < 0.3 indicates weak correlation [17]. We hypothesized that there would be a weak and positive correlation between the total NRS score and the total body area of pain assessed by the pain map, because measures assess different constructs. A significance level of 5% was considered and all analyzes were performed using SPSS 22.0.

## Results

**Fig. 2:**
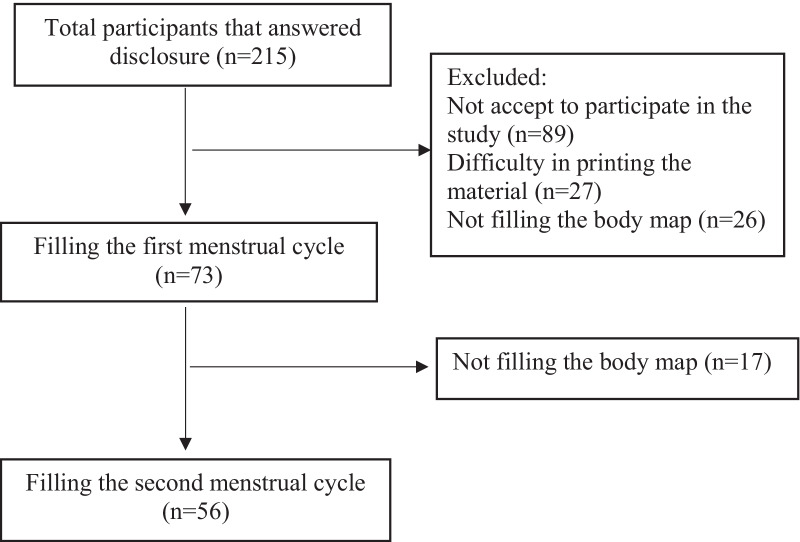
Study flowchart

In this study 215 women answered our disclosure. One hundred fifty-nine women were excluded because they did not accept to participate of this research, answered only about the first menstrual cycle or had difficulties in printing the material or not filling it during the correct days of the cycle (Fig. 2). The final sample consisted of 56 women (24.1 ± 3.1 years old). The mean pain intensity reported by NRS in the second and third menstrual cycle was 6.7 ± 1.8 and 5.7 ± 2.0 points (rho = 0.621; p < 0.0001), respectively. Table 1 shows the sociodemographic and clinical characteristics of PD.

**Table 1:** Sociodemographic and clinical data of women with PD

Sociodemographic variables	Mean ± SD
Self-reported cycle length (days)	28.2 ± 3.8

	n (%)
*Race*	
White	45 (80.3%)
Black	10 (17.8%)
No declaration	1 (1.7%)
*Education*	
Less than college	26 (46.4%)
College	29 (51.7%)
No declaration	1 (1.7%)
*Marital status*	
Single	53 (94.6%)
Married	2 (3.5%)
Divorced	1 (1.7%)

Clinical variables	n (%)
*Menstrual flow*	
Light	10 (17.8%)
Moderate	32 (57.1%)
Intense	14 (25.0%)
*Duration of menstrual flow*	
Up to 3 days	4 (7.2%)
3–5 days	38 (69.0%)
More than 5 days	13 (23.6%)
*Duration of menstrual cramps*	
Less than 24 h	20 (35.7%)
24–48 h	25 (44.6%)
48–72 h	11 (19.6%)
*Use of medication for menstrual cramps*	
Yes	47 (83.9%)
No	9 (16%)
*Use of contraceptive*	
Yes	35 (62.5%)
No	21 (37.5%)
*Effect of medication*	
Total	10 (17.8%)
Partial	30 (53.5%)
No effect	16 (28.5%)
*Body regions with PD-related pain*	
Abdomen	56 (100%)
Low back	44 (78.6%)
Head	31 (55.4%)
Lower limbs	28 (50.0%)
Thoracic region	20 (35.7%)
Upper limbs	09 (16.1%)

Table 2 shows the result of the test–retest reliability between the second and third menstrual cycles, inter-rater reliability of the second menstrual cycle, and intra-rater reliability of the percentage of painful areas in the pain drawing. All the reliabilities were considered excellent (ICC > 0.75) for the total body area of pain. For inter-rater and test–retest evaluations, CCC was 0.96 (0.94–0.99) and 0.71 (0.57–0.81), respectively.

**Table 2:** Test–retest, intra-rater and inter-rater reliability of the total body area of pain in the body map

	ICC (IC95%)	*P*	CCC (IC95%)
Test–retest reliability	0.83 (0.72–0.90)	< 0.001	0.71 (0.57–0.81)
Intra-rater reliability	0.99 (0.99–1.0)	< 0.001	–
Inter-rater reliability	0.91 (0.94–0.96)	< 0.001	0.96 (0.94–0.99)

**Fig. 3:**
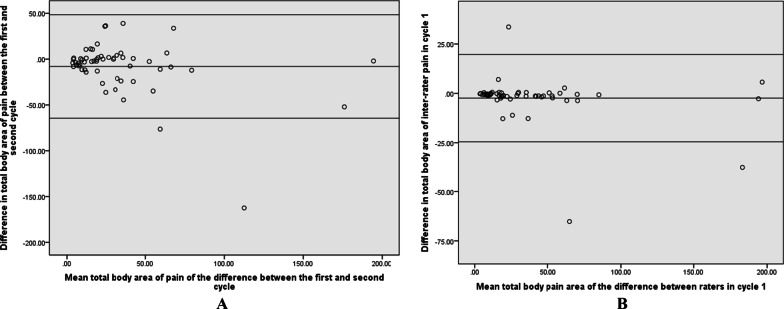
Bland Altman plot for the total body area of pain in the complete sample

The d- between the total body area values of the second and third cycles was − 8.01%. The SEM and SDC values at the individual level were 5.68% and 15.70%, respectively. According to Bland–Altman method, the lower and upper LoA were − 64.52 and 48.50, respectively (Fig. 3a). The d- between the total body area values obtained in the inter-rater analysis of the second cycle was − 2.40%. SEM and SDC at the individual level were 8% and 22.11%, respectively. Lower and upper LoA were − 24.51 and 19.70, respectively (Fig. 3b).

The correlation between total body area of pain in the pain drawing and pain intensity measured by the NRS showed a moderate relationship between these two measures in the first menstruation (rho = 0.30; *p* = 0.021) and in the second menstruation (rho-0.40; *p* = 0.002).

## Discussion

The present study results showed that women have a multisite pain pattern related to PD. The pain drawing is a reliable and valid tool for assessing PD-related pain, with a moderate correlation with the NRS. Excellent ICC values were observed for all reliabilities (test–retest, intra- and inter-rater). Although the ICC values are excellent, CCC was poor for test–retest, but adequate for inter-rater evaluation. For measurement errors, most of the measurements were within the LoA. However, the SDC at the individual level for the inter-evaluator assessment of the second cycle was higher than the LoA of the same assessment, which indicates a real change in the measured construct.

Women who experience PD usually report their menstrual pain occurring on the lower abdomen and pelvis [1]. The present results corroborate those findings, showing that women with PD reported pain in lower abdomen; nonetheless, most of them reported pain in other areas, such as the low back, head and lower limbs, which were present in ≥ 50% of our participants. PD has been claimed to be part of the central sensitization syndromes, and one of the features of those syndromes is the multisite characteristic of the pain [18], which could be found in the present study.

A moderate correlation was found between the pain map and the NRS. This result is beyond our initial hypothesis, as the NRS and the map assess, respectively, the intensity and location of pain. Thus, the pain drawing proved to be valid for the assessment of the location of PD pain. It is possible that women identify greater pain intensity with increasing pain sites, but this is not clear in the literature. To the extent of our knowledge, no studies have assessed the correlation between pain intensity and total area of pain location in women with PD.

The results of test–retest, inter- and intra-rater reliability indicated that the pain drawing can be a reliable measure in women with PD, being useful in identifying the location of menstrual pain and the area of pain affected by different evaluators and times. In addition, it is possible for women to experience different locations and pain intensity of PD depending on the cycle. So, for example, some women may experience less pain only in the lower abdomen, and in the next cycle they may experience more intense pain and pain in other regions, such as the lower back and head. Because of this, it is possible to observe high LoA and occurrence of outliers in the present study. Pain drawings are commonly used to recognize to identify location of pain in different musculoskeletal conditions [19], but there is no gold standard method for measuring painful areas on paper figures [9]. Therefore, it’s very common for these pain drawings to be digitalized using software, as the computer provides a more accurate analysis of the painful area [8]. In addition, some studies have good inter- and intra-rater reliability results in the measurement of painful areas [8, 9, 19, 20].

Regarding measurement errors, the SDC was higher than the SEM, both in the intra- and inter-evaluator analyses, demonstrating the instrument's ability to detect changes in the pain area and the measurement error. The SEM values found corroborate the results of Caseiro et al. [9], who found good to very good SEM values for the pain map in patients with orthopedic or rheumatic conditions.

To the best of our knowledge, this was the first study to assess test–retest, inter- and intra-rater reliability and measurement errors for pain drawing for pain assessment in women with PD. In addition, we used adequate methods to evaluate the total area of pain through health professionals' digitalization of the paper. This reinforces the use of the electronic pain map in clinical practice, especially during remote assessments with patients. In this study, many women had problems printing or filling the Body Map twice; this was a limitation because many women did not have a printer, and some women reported that they answered many surveys during the pandemic period and refused to answer one more.

Nevertheless, the lack of studies on reliability in the use of pain drawings in women with PD limited the comparison of the results of the present study with the literature. In addition, the difficulty in defining analysis methods can impair the clinical application of these pain drawings. In this case, the use of ImageJ as a tool for measuring painful areas proved to be acceptable, since the software helped to identify the specific pain area and spread the discomfort caused by PD in other body areas [8]. Also, this is the first study that has assessed the presence of PD-related pain in different body regions besides the pelvis and abdomen. The other body regions most marked were the low back, head and lower limbs. The second most marked area was the low back, which affected almost 80% of women in the present study, while the head was the third most marked area (55%). Data from the present study corroborates other studies showing PD pain in several different regions [21, 22], indicating the multisite nature of PD-related pain.

Favorable data support the use of the pain drawing for pain assessment in women with PD, considering generalized body pain is a hallmark symptom of menstrual pain, is important its detection in clinical care and scientific research, considering that women with dysmenorrhea could experience restrictions on their daily activities, studies, social and sexual relationships [23]. Physical Therapy interventions or medications can improve pain in a specific area of the body; however, some pain may persist, so a pain drawing that assesses the presence of pain in whole body may be better guide clinical decision-making.

### Implications for practice

The present study shows evidence that supports the application of the pain drawing, commonly used in other painful conditions, as an easy-to-use tool to evaluate the total area of pain of women with primary dysmenorrhea. This leads to a complete clinical assessment of those patients, which involves the site of pain. In addition, we recommend that future studies verify the measurement properties of the body map in health conditions.

## Conclusion

Women with PD present multisite pain and the use of the pain drawing to assess the total body area of PD-related pain was considered a reliable measure.

## Data Availability

The datasets used and/or analyzed during the current study area available from the corresponding author on reasonable request.
